# Informed proxy consent for ancient DNA research

**DOI:** 10.1038/s42003-024-06413-0

**Published:** 2024-07-04

**Authors:** Victoria E. Gibbon, Jessica C. Thompson, Sianne Alves

**Affiliations:** 1 Division of Clinical Anatomy and Biological Anthropology, Department of Human Biology, University of Cape Town, Observatory 7935 Cape Town, South Africa; 2 Department of Anthropology, Yale University, 10 Sachem Street, New Haven, CT 06511 USA; 3 Yale Peabody Museum, Yale University, 170 Whitney Avenue, New Haven, CT 06511 USA; 4 Office for Inclusivity & Change, Office of the Deputy Vice Chancellor for Transformation: University of Cape Town, Ivan Toms Building, 28 Rhodes Avenue Mowbray, Cape Town, South Africa

**Keywords:** Ethics, Evolutionary biology

## Abstract

Embracing the underlying principles and processes of informed proxy consent or relational autonomy consent in human ancient DNA research can transform research.

## Teaser

We argue for implementation of informed proxy or relational autonomy consent in human aDNA research, where the deceased may be represented by living people the research affects. Embracing the underlying principles and process of informed proxy consent has the potential to transform research by (1) enriching outcomes by learning from and collaborating with interested and affected persons; (2) empowering people potentially impacted by research to stipulate evidence for information flow; (3) guarding researchers against actual or perceived violations by providing a common set of guidelines; and (4) highlighting the essential nature of long-term consultation and community partnerships to research outcome success.

Researchers have grappled with considerations regarding the physical treatment of human bodies and their parts (skeletal elements, teeth, hair, nails, soft tissues, chemical, and molecular components), associated cultural sensitivities, the potential for data use and misuse, and the need for collaboration (see examples^1–18^). Even if not legally mandated, institutions and researchers must assume responsibility of ensuring research integrity^10,13,19–21^. DNA from organisms that lived in the past is known as ancient DNA (aDNA), and in specific contexts may be referred to as archaeogenetics. It offers valuable insight into important questions about and applications for human history, illustrated by Svante Pääbo earning the Nobel prize in Physiology/Medicine for his discoveries concerning the genomes of extinct hominins and human evolution^22^. This discipline has undergone exponential growth fueled by technological and methodological developments that open new possibilities for the generation and analyses of data^23–26^. However, drawing on a ‘lag narrative’ whereby ethical and legal considerations that guide research will lag behind rapidly advancing areas of study, does not provide reprieve from responsibility^2^.

Although aDNA is obtained from deceased individuals, it involves biological information that can be *immortalised* in genetic libraries and can impact living people (positively and/or negatively)^1^. These digital DNA libraries constitute the genomic information not only of that person, but also of their ancestors, and may be stored for an unforeseeably long time. Therefore, ethical guidelines appropriate for this work must protect past, present, and future generations. The ‘Singapore Statement’ is a global statement on ethical research that acknowledges that different social, political, cultural, and economic contexts prohibit generation of a universal set of specific guidelines. However, four principles apply: honesty, accountability, professionalism, and stewardship^27^.

To allow for global flexibility, for the purposes of this paper we use ‘interested parties’ to denote individuals, groups, or other parties who may be affected by the research. A non-exhaustive potential list includes people who identify as part of culturally and/or biologically descendant communities; caretakers of cultural knowledge who may or may not claim direct descent; people who live near or identify origin from the location of remains; community leaders, elected or appointed officials at different tiers of government (who may not be involved in the consent process but would be engaged with formal written applications and permits); researchers who have previously studied the individuals or communities; and institutions responsible for the stewardship of remains. We do not claim that all interested parties should have equal prioritisation in the consent process. Descendants are referred to those who share genealogical or cultural history relationships with the ancient individuals being studied and may also be caretakers of cultural knowledge.

The need for use of specialised aDNA laboratories, most of which are in the Global North, increases the risk for *parachute research* and/or *ethics dumping*^28–32^. Parachute research occurs when researchers based in a well-resourced place do research in a less-resourced place, make use of the local infrastructure and personnel, obtain samples, and then leave without returning or communicating results to interested parties, usually those at the local level. Ethics dumping is when researchers go into areas with less research regulation, oversight, and ethical standards to do research in a manner that does not meet the ethical standards of where they reside. Steps to reduce these extractive practices are required but are highly context-specific, and there is no one-size-fits-all solution^9^. However, as previously noted there is a need to develop guiding principles that can be applied across the aDNA community of research^13,17^. One area such principles can be further developed in detail, and which does more to include people beyond those identifying primarily as researchers, is how to obtain consent.

The concept of obtaining permission in aDNA analogous to *proxy* informed consent, as is obtained from living people, was discussed by Holm^33^ and Kaestle and Horsburgh^11^. Both rationalised reasons to disregard the concept for ancient tissues. In these arguments, they do not recognise the potential impact for living descendants even if distant temporally, spatially and/or culturally. Fleskes et al.^17^ reviewed ethics in aDNA research, and found that more than twenty years later consent is still not universally mandated, nor typically obtained.

The process of obtaining informed consent reduces risk in aDNA research as it necessitates engagement with a range of potentially interested parties, sharing genuine authority in the research by providing space to detail the conditions whereby they agree or disagree for research to occur. Improving consent rigor may partially address inequities and inherent power imbalances between researchers and other interested parties, particularly if researchers are not from the communities most likely to be impacted by the research. It is also likely to enrich the research results through new perspectives, information, and ideas.

Here, we present considerations for undertaking a version of informed proxy consent modified for aDNA research. We illustrate how principles often employed in a consultation context can be applied to a more explicit consent process, thus establishing long-term, multi-staged relationships with communities that anticipate continued engagement beyond a single research study. We also show the applicability and feasibility of obtaining informed proxy consent within an ethics-of-care framework outlined in the *relational autonomy* approach^16,17^.

## What is informed consent?

Informed consent, as summarised from the Declaration of Helsinki^34^, requires those asked for consent to be adequately informed of the study aims, methods, sources of funding, conflicts of interest, institutional affiliations of the researchers, and anticipated benefits and risks of participation. It also requires that participants provide their consent without coercion, understanding they can withhold or withdraw it at any time^34^. The World Health Organization^35^ adds that ‘research has impacts not only on the individuals who participate, but also on the communities where the research occurs and/or to whom findings can be linked’ pp.14. Therefore, researchers should actively engage with communities in decision-making regarding research design and conduct to ‘minimise negative effects on communities’^35^ pp.14.

Informed consent comprises two core concepts: (1) those who provide it have an appropriate level of capacity/competency and access to adequate information they require to comprehend the implications of their participation in a study, and (2) voluntariness focused on decision-making free from coercion or undue influence^36,37^. While researchers may also be from and/or of the community under study, their specialised expertise affords them greater access to information about a study and its broader scientific implications than most descendants and other interested parties from the community. Therefore, researchers are positioned to intentionally or unintentionally withhold key information regardless of their individual backgrounds. These compound the potential barriers faced by descendants and other interested parties in their ability or willingness to raise questions or concerns (e.g. language differences, previous exposure to the research topic, or cultural differences in the expression of dissent). The onus is for *researchers* to document the steps they have taken to assure understanding, which is critical to validate consent^38^.

The concept of informed consent is a fundamental principle in research involving living humans. However, its application especially to the deceased lacks consensus, positioning it delicately within a growing conversation regarding how guidelines developed under one paradigm may not be directly transferable to others (Table 1)^35,38–41^. The dominant philosophical basis of the process of informed consent is grounded in a cultural context that prioritises the will and autonomy of the individual, and its implementation is often conducted in writing^17^. However, this foundation is not always appropriate for societies that vest the responsibility of decision-making in communal authority, and/or where literacy is limited^35,41^. There is a need to develop more flexible, context-specific, and culturally appropriate ways of obtaining and documenting consent^42,43^. The process of consent should be scientifically rigorous, legally robust, ethically sound, and practical to obtain, all while recognising that due to context specificity no universal definition of these can exist^18,21,36,42,44–50^.

**Table 1:** A comparison of informed consent procedures for research on living humans with that of deceased individuals in ancient DNA

	Proxy informed consent in ancient DNA research	Informed consent with living people
Consent provided by	Representatives of interested and affected persons, groups and/or parties.	Individuals.
Consent provided on behalf of	Past and future generations, living communities.	Individuals, with population-wide implications.
Where consent process begins	Descendant(s) and community interested and affected persons, groups and/or parties.	Most local and international ethics review boards.
When consent process begins	During initial community consultation.	After research is approved.
Legal obligations for consent	Some local and international ethics review boards.	Most local and international ethics review boards.
Location of research samples	Often away from impacted communities.	Varies; can be local, national, or international.
Urgency beyond research needs	Situationally dependent, may not have an urgency, or may be used for restitution, land claims, conservation, or to address stigma. conservation,	May address urgent or time-sensitive health or economic issues.

## Proxy consent in aDNA research

In cases where a living person is unable to consent to their own participation in research, proxy consent can be obtained from an individual or group of individuals who assume the responsibility of decision-making on their behalf^35,51–54^. Among the living, there are legal options that allow an individual to appoint a specific person (e.g. relative, friend, or caretaker) to make decisions and provide consent. If such a person is not appointed, the legal authority for the deceased is often their next-of-kin, who may be able to overwrite decisions requested or stipulated by the decedent^36,44,55^.

While the existing framework of considerations for conducting genetic and genomic research ethically in living people can be (and has been) used as a guide for articulating considerations for aDNA research^17,39^, aDNA research faces a complex situation because consent from the decedent is impossible. Individuals under study are often many generations removed from living people, some with stronger connections than others. Considering this, we draw on the concepts of proxy and *relational autonomy* consent (see Table 1). Informed proxy consent in this context is where a person or group of living people are asked to provide permission on behalf of a deceased person. Where people are not culturally considered to be fully autonomous individuals, which are concepts rooted in Western perspectives of the deceased, individuals can be viewed as embedded in communities where legitimate authority to consent is ‘situated in historical, social, class, race, and gendered contexts’^53^ pp. 376. Thus, proxy consent usage is more akin to the concept of relational autonomy-informed consent (i.e. ethics-of-care framework)^53^. Relational autonomy consent may not currently carry identical legal ramifications as informed consent with living participants, but it does place a clear obligation on researchers, institutions, and publishers who use research results to articulate and adhere to the wishes of interested and affected parties^7,8,17,26,40^.

Two salient practical complexities exist when considering how to integrate the concept of relational proxy informed consent into aDNA research. The first is how to identify the relevant descendant communities and/or other interested parties appropriately and exhaustively who have the ethical mandate and/or legal authority to make decisions on behalf of the deceased. It is important to recognise that both kinds of authority may not be vested within the same entities, although identifying the latter may be a more straightforward process. A key reason why it is important for the aDNA research community to adopt a process of consent is because it has a history of conflating the ethical and legal mandates, with legal permissions being prioritised or considered sufficient on their own^14^.

The second complexity is one of enforcement. Research on living people or the recently deceased is almost universally regulated in some way that involves informed consent. These regulations operate at multiple structural levels, and many are legally enforceable. These include legislative inspectorates; institutional review boards and ethics regulatory committees based at institutions; requirements by funding agencies before they provide support; and academic publishers that require evidence for compliance of study prior to publication. The aDNA research community has the power to proactively counter the ‘lag narrative’ by adopting standards that may not—at least at first—be legally mandated, but which are nonetheless effective at ensuring due consideration is given to how consent is obtained, and from whom.

These complexities are similar to the issues faced by researchers wishing to study any biological tissues from a deceased person^56–59^. This large literature is impossible to detail here and is an ongoing discourse. Even so, the framework we propose contains two practical elements that will directly improve the ethical rigor of the entire process. The first is that integrating a consent process into aDNA research as a community standard motivates researchers to articulate how they obtained consent, including how they arrived at determining which entities could provide it in the first place. It highlights and opens to scrutiny information about research design that is often hidden or assumed. The second element is that by emphasising the ‘informed’ aspect of consent, researchers are urged to be transparent in their communication to those providing consent about the limitations of what they are requesting. For example, the documents that people sign may not be legally binding in every context, although they may offer evidence in a legal dispute. Providing this information as an integrated part of obtaining consent lowers the risks of misunderstanding and conflict later in the research process.

## Proxy consent as a process

For the remainder of the paper the term ‘consent’ implies it is informed and ‘proxy’ is relational autonomy proxy. Following consultation, involved community members may understand what a researcher wants to do with their data for a specific project. However, to adhere to the World Health Organization and Helsinki Declaration policies for research consent, entities providing consent must also be adequately informed with comprehension of how data may be used after project completion^34,35^. For example, when DNA is extracted, it is converted into digital and physical DNA libraries, which are *immortalised* and *accessible* records of the original sample. These libraries are an important archive for research, allowing study replication. However, for some interested parties, the preservation of biological material can be inherently antithetical to the natural and universal experience of death^60^. Understanding how long data derived from a study may live on after living people who provided consent are gone may not always be known, nor foreseen^61^.

Digital and physical libraries can also be repurposed for new studies that were not considered possible during the initial project design (e.g. Havasupai Case^16^)^17^. Currently, most published genome sequences become part of an openly available digital public record, which can be used in different and possibly unanticipated ways far different in scope and intent from the original work^17^. While the obligation to conduct transparent and replicable research is foundational to the scientific process^13^, it is equally incumbent on researchers to protect data from being deployed in ways beyond what is consented to or comprehended by interested parties. Treating consent as a process, rather than a single contractual moment, provides space for these considerations^62^.

Consent involves a commitment to continuing conversations that bring descendants and other interested parties into the research process from inception to completion and, where possible, empowers them to determine future knowledge mobilisation. Such an approach would reduce consent retraction, as the research is mutually beneficial and understood. A useful approach for aDNA is known as *dynamic consent*, a personalised approach involving bi-directional regular communication between researchers and participants^63^. Consent is both a research journey and an opportunity to cultivate an equitable partnership where descendants and/or other interested parties can enrich research with the questions and perspectives they are in unique positions to ask and offer. It is an opportunity for researchers to hear the needs of people outside their immediate academic circles and understand their desired outcomes as the research is designed.

## Considerations for proxy consent processes in aDNA research

**Fig. 1:**
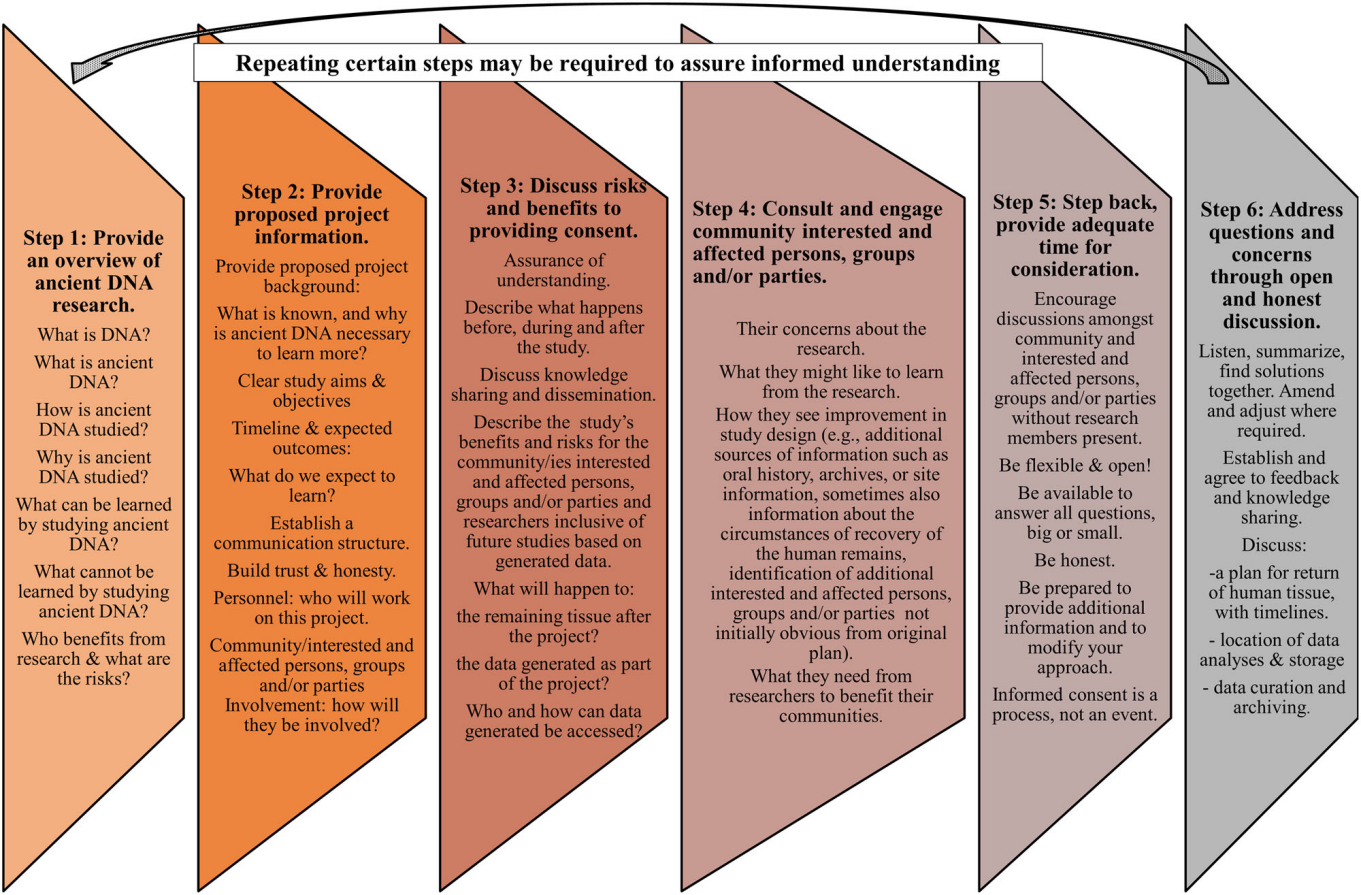
A step-by-step summary of considerations for achieving a process of informed proxy consent for ancient DNA research.

Differences between the process of consent in research with living and deceased humans are summarised in Table 1. Tables 2–4 provide a series of considerations for engaging in a process of consent for aDNA research, and Fig. 1 is a schematic summary of the consent steps.

**Table 2:** Initial considerations for a process of informed proxy consent in human ancient DNA (aDNA) research, aligned to Fig. 1 steps 1 and 2

**Project overview**
What are the scope and intentions of this research?
Why is this research important?
What research questions will be investigated?
What value would aDNA add to our understanding? How will aDNA expand on what is already known?
Where will the research be carried out?
Who is funding, or potentially funding, the research?
Who will work on the research? Disclosure of personnel. Define each person’s role.
What analytical techniques will be used?
What is the expected impact on the human remains? How much and what tissues will be used?
What type of data will be generated?
How, where, and by whom will results and data be stored and disseminated?
What are potentially sensitive topics and aspects of the study?
What are the expected results?
How can the project be revised or changed based on community requests?
What is the project timeline?
Are there plans to expand the project? If so, what will additional consent and engagement processes involve?
Document the process from the beginning and throughout its duration.
**Limitation of study aims & objectives**
Are the study questions and scope clearly defined?
What are the benefits and significance of this research for descendant(s) and interested and affected persons, groups and/or parties from the relevant communities?
What are the risks of this research for descendant(s) and interested and affected persons, groups and/or parties in the community?
How do the researcher(s) and institutions tangibly benefit from this research? What’s in it for them?
How do the descendant(s) and other interested and affected persons, groups and/or parties benefit from this research? What would they like from the research and why would they like the research done?
What has been implemented to ensure protection that researcher(s) cannot use these samples for other research questions and studies not approved through this informed consent?
**Communication structure**
Identify a representative or group of representatives from the descendant(s) and/or interested parties who can act as a broader liaison(s).
Identify accessible communication tools (Email, WhatsApp, Teams, Skype, Zoom) etc…
Are there language barriers between the research team and descendant(s) and community interested and affected persons, groups and/or parties?
Has a nominee been nominated and agreed with consensus to carry voice of descendant(s)/community interested and affected persons, groups and/or parties?
How will decisions be made? When can the appointed contact person make decisions and when will broader consultation be required?
Is there a plan for capacity building or training?
Provide time for thoughtful consideration and engagement of ideas.
Develop a plan for engagement throughout and beyond the study to maintain relevant communication channels.
Awareness that back-and-forth communication can be slow, factoring in reasonable time.
Identify a plan for obtaining and executing research consent.

**Table 3:** Considerations regarding assurance of understanding towards building trust and honesty for informed proxy consent in ancient DNA (aDNA) research, aligned to Fig. 1 step 3

**Assurance of understanding & comprehension**
Consent requests should be direct, honest, and respectful.
Conduct the initial meeting without intention to carry out any work.
Forms and documents should be provided in the home language of descendant(s) and interested and affected persons, groups, and/or parties from the community.
Forms and documents should be provided orally, visually, and in writing.
Hold a question and answer session immediately with a second meeting days or weeks later to allow for full consideration and further questions to be raised.
For independent research and to substantiate legitimacy, provide additional readings, websites, and names of researcher(s) who will be involved.
Documentation should be prepared in non-scientific, simple, and clearly understandable language and comprehension (recommended 6th to 8th grade reading level).
Descendant(s) and interested and affected persons, groups, and/or parties from the community can dictate, provide a visual representation, or write out in their own words and language from their understanding what is agreed to and not agreed to. Add these as an amendment to the consent documents. Identify if there is interest or objections to this?
Are any of the research team from the country/region of origin?
Have the correct local rules and laws been followed?
Have the researcher(s) consulted local heritage authorities to identify the requirements?
Ensure there is dialogue amongst all researcher(s) who will access the human remains or data from the project, to articulate that they are committed to respect and honour agreements.
Are all researcher(s) involved in discussion with descendant(s) and interested and affected persons, groups, and/or parties from the community?
Researcher(s) contact information should be made available. Answer questions within a week.
Listen carefully to the concerns of the descendant(s) and interested and affected persons, groups, and/or parties from the community.
Provide time for open conversation, discussion, and question asking/answering.
**Building trust & honesty**
Keep promises.
Be transparent.
Respond timeously to queries.
Provision of regular updates on the study.
Be honest of outcomes and challenges.
Acknowledge study permission is a sharing of stewardship and custodianship for the human remains and their data.
Ensure data ownership is clear to the descendant(s) and interested and affected persons, groups, and/or parties from the community, and that there is good faith effort to place as much data ownership and control into their hands as is legally possible.
**Knowledge sharing & dissemination**
When can results be reasonably expected?
How will results be shared? With and by whom?
Who will interpret the results? Is there a role for descendant(s) and interested and affected persons, groups, and/or parties from the community in the interpretation?
What happens if the results are negative or fail? Will these still be published and shared?
Clarity on academic/scientific goals and benefits of this project e.g. publication, presentation at conferences.
Honestly communicate possible outcomes, recognising that the analysis of genetic data can lead in unanticipated directions.
Disseminate results in both academic and non-academic outputs, ensuring accessibility to the community.
Ask how the community would like the results and information sharing to occur.
Is there a role for consortium style authorship or acknowledgement available for the descendant(s) and interested and affected persons, groups, and/or parties from the community? Have the risks and responsibilities of this been disclosed and discussed?
What recourse do descendant(s) and other interested and affected persons, groups, and/or parties from the community have to claim or rescind research/output that is generated?
Annual updates on progress.

**Table 4:** Considerations regarding the storage, curation, access, and archiving of samples/data for informed proxy consent in ancient DNA (aDNA) research, aligned to Fig. 1 step 3

**Access and return of the physical human remains**
Will a materials transfer agreement be implemented, and on what timeline?
Who will have access to the biological and physical samples and for what purpose(s)?
What assurances do the descendant(s) and interested and affected persons, groups, and/or parties from the community have that all physical biological materials will be returned as agreed?
How would the descendant(s) and/or interested and affected persons, groups, and/or parties from the community like the unused samples/physical human remains to be handled, imaged, and/or displayed in publications or presentations? Discuss feasible options.
**Location of data analyses & storage**
Where will the aDNA research be conducted?
By whom will the aDNA research be conducted?
Who is the principal investigator responsible from those locations? What have their relationships been with other communities/interested and affected persons, groups, and/or parties in the past? Do research.
Do they have good track records of following through on community requests?
Discuss access and return of unused human remains, including a timeline.
Discuss storage or destruction of liquid DNA and DNA extraction products (including synthetic libraries).
Discuss data storage and sharing.
**Data curation & archiving**
Data are inclusive of the entire study process not only the DNA results. They include full documentation of the study and process including decisions, minutes and consultation outcomes, signed consent documents, and any photographs or videos taken during the process.
Discuss data curation and accessibility during and post-study. What happens after the study?
Where will all the data be stored?
Who will have access?
Will an archive be created? If so, where and what are the access requirements.
If people want to access these data for another study what is the process? To what extent will the descendant(s) and/or interested and affected persons, groups, and/or parties from the community be notified, informed, and have a say?
Create a permissions and restrictions document the following types of research can be done without further permission and those that require permission.
Copies of all outputs should be sent to the descendant(s) and interested and affected persons, groups, and/or parties in the community, or to their nominated liaison(s).
Where possible, information should be provided in lay summaries in their home language(s).
For transparency a document should be circulated with exact locations of all data related to the research.
Notification via email being required for all research set limits such as: with 30 days to respond.
Provide the opportunity for descendant(s) and interested and affected persons, groups, and/or parties from the community to state what is not allowed, e.g. no images of human remains may be used in publication.
Descendant(s) and other interested and affected persons, groups, and/or parties from the community should be notified of any further research using the data, within a timeline allowing space for response.
Descendant(s) and other interested and affected persons, groups, and/or parties from the community should be provided the opportunity for a new consent process for additional research, or to specify the types of research that may proceed under the current consent.
Descendant(s) and other interested and affected persons, groups, and/or parties from the community should outline for future research and all stemming research how and if they want to be mentioned and acknowledged. This can be done by creating an acknowledgement statement for future studies.

Notably, individuals providing consent generally do not act as a direct proxy for an individual but are acting in relational autonomy for entire groups of people who may span generations past and present. The strength of linkage to the ancient person/s by potential interested parties will vary enormously from context to context. Navigating these relationships, and identifying representatives of interested parties, often occurs through a process of consultation, which is legally, culturally, and regionally situation-specific^7,8,13,17,18,26,37^. Due to the situation and context specificity of identifying representatives, which is described in the cited references, we provide guidelines here for the consent process after appropriate descendants and/or interested parties have been identified.

### Initial considerations

Consent as a process begins during the planning phases of a research project and incorporates iterative feedback from descendants and other interested parties into the research design. It starts by developing a draft work plan that identifies the study intent, aims, and importance, understanding that the specifics or underlying questions may require revision during the consultation and consenting processes. As researchers gain from the work, they should consider during consultation how the research can benefit people locally^3,23^. They should assess if and why aDNA is necessary to inform this project and use available experience to assess potential risk. For example, might aDNA suggest one group of people has a stronger claim to land over another? If there is a chance of harm to any living person or group, the research should be reconsidered in accordance with the concept non-maleficence (no-harm) as prescribed in the Belmont report^64^.

Next, researchers should develop a detailed project overview (see Tables 2–4), including (but not limited to) information about who will participate in the research and in what roles, as well as details of the necessary materials, analytical techniques, and plans for data generation and storage. This is another point at which researchers can reflect on their positionality and goals relative to other interested parties to ensure the plan is sufficiently detailed for clarity and adaptable to community feedback. Plans should be aligned to the laws, rules, and regulations for the country and local regulatory authorities where samples will be obtained. To actively counter the ‘lag narrative’, researchers should lead with ethical considerations rather than fulfilling minimal requirements. This includes being mindful of not perpetuating parachute or extractive research and taking demonstrable steps to ensure inclusivity and research integrity. As described previously, there may be gaps in laws and regulations, and it is for researchers to identify and bridge them^10,18,26,61^.

It is imperative that information is relayed in a manner appropriate for an audience who may not have a deep knowledge of the subject matter. When developing a process of consent, a researcher should not make a priori assumptions about the education or scientific/research experience of the descendants or other interested parties. In this regard, they are typically in a position of greater vulnerability relative to the researchers. Information should be communicated in the home language/s of the descendants or other interested parties, and optimally, via multiple formats of delivery (visual, oral and written). As a guideline, pamphlets with information written at a reading comprehension level of sixth to eighth grade should be provided^65^, given cross-cultural differences in formal literacy acquisition and use. It is the responsibility of the researchers to provide the necessary information regarding the project, and also its potential outcomes and impacts. This information should be conveyed in a way that is community-centered rather than researcher-cenetred, i.e. in a way that maximises comprehension even if it has the potential to raise concerns. It is also important that researchers understand that comprehensible information is not synonymous with comprehended information and incorporate tools for checking that comprehension has been achieved.

Researchers should support local involvement in the development of communications, for example, by enlisting and compensating a local community member for translation^39,41^. They should seek aid from people who can transform research objectives into culturally relevant metaphors or develop helpful multimedia tools to ensure complex concepts are understood^45,66,67^. Hands-on activities can be used to investigate comprehension (e.g. encouraging people to describe a part of the research in their own words). Advance discussions are crucial tools to ensure understanding in a way that acknowledges literacy and language levels and acceptable modes of consent^42^. As a lengthy and detailed document may fail in the objective of being readily understood, this process will enable the consent document itself to be designed in the clearest and most succinct and culturally comprehensible manner^68^.

The format of communication during the processes of consultation and consent is context dependent. Large-group engagements may fit some settings, while conversation with a liaison or a small advisory group appointed by the descendants and/or other interested parties may better fit others (see^20^ for suggestions). The researcher must provide time for discussion between interested parties without researchers present, and they should provide necessary and reasonable resources for the execution of these processes. Importantly, the larger community of research (e.g. representatives of institutions, funding agencies, colleagues acting as reviewers of grants or academic appointments) must understand this process to be an essential element of research that can rightfully require time and financial resources to execute.

We further recommend researchers discuss and consider benefits to the community beyond the study. Funders and employers also need to understand this is essential for ethical research. For example, VEG in South Africa provides lessons to secondary school learners on the human body and raises funds for education outreach (classroom furniture, teaching supplies, and books) in the communities she works. JCT in Malawi has invested in public information days, community workshops, documentaries in local languages, hire and involvement of community representatives, and repairs to the community heritage centre. These are immediate ways of demonstrating investment, understanding, and tangible benefits for communities.

### Community-engaged research design

Grounding processes of proxy consent in community-engaged participatory research design is ideal, but not always possible. An ethics-of-care framework requires good faith efforts to identify interested and affected persons, groups and/or parties, with robust consultation as the foundation for all that comes after. In biological anthropology and aDNA research the importance, value, and strategies of community partnerships in research have been well described, along with the context-specificity of how to identify descendants and other interested parties, and the associated challenges of this work^7,8,18,19,37,69,70^. We describe the consultation process here in the context of developing a pathway to relational autonomy proxy consent, remaining mindful that as the consent process unfolds, unforeseen interested parties may emerge. We acknowledge that this kind of consultation may demand additional and often challenging work, especially relative to academic expectations for a fast pace of research publication, but it is also both ethically robust and an opportunity to uplift communities on which research results can depend.

During consultation, researchers should be mindful of diverse perspectives regarding scientific research. Interest from well-resourced external entities can become politicised and exacerbate existing tensions, so researchers should consider the impacts of different modes of consultation, and who might feel excluded under each. In some contexts, an obviously foreign or unfamiliar researcher appearing in person as a first step may create discomfort, giving the impression of seeking a business transaction rather than partnership, or it may raise suspicions that there is an economic benefit for only some community members. Conversely, in some contexts it may be construed as rude or suspicious if no initial in-person appearance is made by the lead researcher. It is important to work closely with local collaborators from project conceptualisation, as they can share guidance surrounding cultural norms around whom to contact and in what sequence.

Part of transparency means discussing previous research that may be of relevance to the group. Differential access to resources can render descendants and other interested parties unaware of the existence of, or ability to access, collections, manuscripts, and other sources of data held in archives or behind paywalls^69^. If such work was executed without consultation, consent, or return of results, the community may be surprised or offended. It is important to discuss how the proposed research will not recapitulate problematic practices and narratives that community members have already experienced. Researchers should then be prepared for multiple iterations of consultation during the consent process.

Figure 1 is a schematic of the steps during consultation, development of research design, and consent. Community engagement and partnership during project conceptualisation provides a forum to reveal new perspectives, sources of information, and potential pitfalls. This consultation phase may or may not require permitting as per local requirements (i.e. ethics, administrative, and/or regulatory).

When there is consensus regarding the final proposed study design, it is appropriate to apply for additional funding or permissions to obtain consent. The project may now look quite different from what was originally envisioned. In addition to communities having certain standards about the research, scientists also have a code of scholarly ethics. Any party should feel they can walk away from the process at any time without prejudice, taking steps to foresee that at an advanced stage of the study this will be practically more challenging. Due to past injustices with scientific research, we implore researchers to limit their study design and consent to well-defined and meaningful questions. Obtaining consent for a limited set of questions and a singular study creates the need for researchers who want to utilise data in new ways to seek consent for a follow-up or newly conceptualised study. Researchers should also ask, rather than assume, the situations under which those providing consent would desire this to occur, similar to the concept of ‘meta consent’^71^.

### The consent process

The steps in Fig. 1 apply to both the research design and consent phases, and we describe them here, with considerations for execution presented in Tables 2–4. Figure 1 steps 1 and 2 can be achieved with and correspond to Tables 2 and 3.

Step 1: Provide an overview of aDNA research, with an explanation of what DNA and aDNA are, how they can be studied and what can be learned by studying the genomes of past people, and the possible risks and benefits of aDNA research. It is important to offer both introductory-level and (if appropriate) more in-depth explanations in written and/or visual form (e.g.^24^) to provide participants with something tangible and permanent to review after the research team has left (Table 2). It can also be shared with people unable to attend the meeting and provide a starting point for questions during subsequent consultations.

Step 2: Provide a background to the proposed project about why aDNA is necessary to learn more and explain the specific study with an outline of discreet aims and objectives (see Table 2). Explain the timeline and expected outcomes, establish a communication structure, and build honesty and trust. Describe the team and personnel, with an explanation of how the descendants and/or other interested parties will be involved. Document the consultation.

Step 3: Discuss the risks and benefits of the proposed work. Explain what happens before, during, and after the study (Tables 3 and 4). Set timelines and develop a reliable system of communication for updates. Discuss how knowledge generated by the study will be shared beyond the scientific/research community, especially to the descendants and other interested parties. Ensure that there are clear expectations regarding the mode of information transfer, to whom, and on what anticipated timeline (Table 3). It is similarly important to be honest about the potential risks of fully open access data, including how rapid advances in data analysis may result in uses for the data that cannot be fully anticipated in the present day.

As research is not selfless, explaining the academic benefits to the researchers and their institutions is important (e.g. research for degree purposes, research outputs, or applying for promotion) (Table 3). The institution and researchers benefit indirectly by knowledge production, gaining visibility, credibility, and recognition for their contributions in the academic sphere (research rankings). They may benefit from promotion, media, and publicity. There can be financial benefit for the institution and/or researchers through grants, book royalties, awards, and job retention. Use follow-up questions or ask community members to state what they perceive the benefits to be, to gauge assurance of understanding and allow for articulation of why those consenting would be interested in the research, and what they might like to do with the research findings (Table 3). This engaged scholarship approach provides time for reflection and the opportunity to express the value of this research process/output from their own perspective^7,17,26^. Consideration should be given to the weight of the benefits to researchers versus those for descendants and other interested parties, again with a mind to the principle of non-maleficence (no-harm).

Risks for descendants and other interested parties can come after providing consent. Discuss who will have access to the physical human remains and if and how any unused sample material will be returned (and if so, to where, by whom, and on what timeline) (Table 4). Discuss how unused samples/physical human remains and by-products (i.e. extracts, DNA libraries, CT scans) will be curated after sampling, and present some feasible options (e.g. in the South African Sutherland process all by-products from the sampling, including the aliquots, were returned for reburial^15^). Together, arrive at a data curation and archival plan inclusive of documenting the consent and any applicable ethics permissions, and assure that local communities are comfortable with it (Table 4). As is recommended for living persons, and where possible, these aspects can be covered in a materials transfer agreement between institutions with responsibility to safeguard the interests of interested parties under their authority. These protect all parties involved^35^. This is especially important where samples or data will be exported out of country, as they are binding beyond the lifetime or employment status of an individual person.

Researchers can include in the consent process ways to demonstrate respect for the contributions of those providing consent as knowledge-holders (Table 3; see^17^ Fig. 1 pp. 645). Examples can be a consortium authorship or a more general acknowledgement statement. All those who make an intellectual contribution should be engaged regarding co-authorship^8,26^, considering that intellectual contributions may require a more inclusive definition when research is co-created across a broad range of interested parties. Authorship is widely defined by institutions and journals, and not limited strictly to individuals with certain degrees or who directly participated in writing or analysis phases of research^19^.

Step 4: Consult and engage descendants and/or other interested parties on their concerns about the research, and what they might want to learn. Provide an opportunity for new insight into the study design and ask them identify ways they may see the research as beneficial beyond the benefits apparent to the researcher.

Step 5: Provide time for descendants and other interested parties to discuss the research proposal and the request for consent. Researchers need to be aware that their physical presence in a space invokes power dynamics. The existing power differential between the researchers, who are motivated to obtain consent, and the persons providing it, can also be exacerbated by structural coercion, wherein people feel compelled to provide consent because of their position in the larger socioeconomic system^61^. Therefore, this step is about providing adequate time for consideration of the information and request. A useful approach is to designate a local liaison to consult with the community or operate as a note-taker and provide a list of questions and concerns for the researchers to address when they re-enter the space. This may not necessarily be on the same day, so ensure there is information about when researchers will return to answer questions and address concerns. Recognise that descendants and other interested parties may not be ready to provide answers on the timeline prescribed by the researcher(s).

Step 6: Re-enter the consultation space to listen, summarise, and find solutions in the study that are acceptable for all parties. This process should be documented to the level of comfort dictated by the individuals with whom researchers are consulting, and it should be clear in advance where any notes or recordings will be kept and who will have access. A data management plan to document the process of consent and research partnership will be important to clarify agreements. This is the phase of establishing an agreement for signature and formal consent. If documents require modification, this may initiate a need to submit revised versions to regulatory authorities and returning to the community later for further consultation before obtaining signatures.

It may also be the case that potential interested parties elect not to participate, or do not wish to provide either consent or dissent. They may not feel comfortable deciding on behalf of the decedent, or they may feel they are not the appropriate people to do so. It is still important to document the process by which they received information and elected not to participate, by formalising on a document that they chose to abstain. This process of ‘informed abstinence’ may appear very much like the process of informed consent described above.

Supplementary information is based on an example implemented by author JCT, which was used as a summary of the kinds of analyses that could be applied to ancient human remains, associated risks, including the likelihood that they will actually generate or not generate the desired results, a description of the necessary types of analyses, level of destruction, and data generation, and space to append details of how samples should be handled, curated, and disseminated. This document was introduced after initial community consultation and modified over multiple days under further consultation between community members, researchers, and government representatives. It is offered here as a baseline template for how a consent document might appear and shows how context-specific each situation will be. Its inclusion is to demonstrate how consent for studies of ancient people can be formalised, and it offers an adaptable starting point for conversations between researchers, descendants, and other interested parties.

After consent is obtained in Step 6 and relevant permissions are implemented, sample acquisition, processing and analyses may proceed. During this phase, researchers should provide regular progress updates; we recommend contact at least twice per year or as major milestones are reached. We suggest results be shared with descendants and other interested parties who provided consent before being prepared for publication, allowing for insights and an opportunity for interpretation and preparation for public outcomes^7^. After acceptance of a publication, these same representatives should be contacted in advance with any press releases or other public-facing products. This process also requires informed understanding (repeating the steps in Fig. 1 may be required). For some aspects of the study, the process of consent may occur in perpetuity, meaning a long-term partnership of continuously revisiting the consent process as data are revisited, and/or policies evolve (e.g. data curation, access, relocation, physical commemoration, etc.).

## Discussion and conclusions

Some researchers may consider extremely ancient human remains as outside the need for consent other than destructive sampling permissions from the curating institution, e.g. Neanderthal remains that are tens of thousands of years old. However, some cultural groups strongly identify with ancient people regardless of the temporal distance. For example, Australian Aboriginal communities around the Willandra Lakes region have demonstrated strong cultural affinity to human remains, resulting in successful repatriation requests which are of similar antiquity to some sequenced Neandertals^72^ Both biological and cultural connections can endure for extremely long periods (e.g. Kennewick Man/the Ancient One^73^). Therefore, time-since-death or ancientness alone are insufficient to reject the need for good-faith efforts at obtaining proxy informed consent (or informed abstinence) beyond the curating institution, using the model of relational autonomy discussed above. This is especially important for aDNA research that targets samples from Indigenous ancient people whose descendants may be vulnerable or marginalised, as was discussed at length during the human genome project and human genome diversity project^74,75^. We acknowledge that all interested parties’ claims have the potential for entanglement with political ideologies and that assigning ‘legitimacy’ to claims is in and of itself rooted in power differentials. The key point is that seeking proxy informed consent beyond the curating institution may be appropriate even where there are no clear legal requirements to obtain it. The aDNA research community and other interested parties all stand to benefit from documenting and conducting proxy consent as a routine part of how research is conducted and reported.

Where possible, the consent processes should be entered with an unwavering commitment to ensuring that the descendants and other interested parties are thoroughly informed on all aspects of the proposed research, including the reason for the research, the analytical methods to be used, the potential risks and benefits to the researchers and to themselves, the expected outcomes, and the way the results will be disseminated. The consent process should incorporate procedures to empower the people providing consent to foster their inclusion in the research design and use of research outputs. This includes an active disinvestment of power by researchers that allows descendants and other interested parties to provide guidance regarding how they wish to benefit from, contribute to, and use the information that is generated. Though there are core aspects that remain the same, the process of obtaining consent can (and must) be adapted for appropriateness for each sociocultural and regional context. The World Health Organization states ‘researchers should actively engage with communities in decision-making about the design and conduct of research (including the informed consent process), while being sensitive to and respecting the communities’ cultural, traditional and religious practices^35^. pp.15’ In the Sutherland Nine^15^ restitution process in South Africa, with authors VEG and SA, the descendant community conveyed their wants, expectations, and restrictions written in their own words and home language, which was added as an appendix to the consent process.

Structuring the consent process to invite conversation, dialogue, historical sharing, and to ensure that the knowledge is co-developed and designed, offers reciprocity to community participants, especially because knowledge transference does not have the same impact (positive or negative) for a community as it does for researchers/institutions. A key example is the intersection and sometimes direct conflict between the needs of communities to protect and control data access and increasing calls for open access data in aDNA to facilitate replication of results^1,14,66,70^. One way to mitigate these risks is to limit data usage to specified study aims and objectives, with clear stipulations about future usage restrictions. While it does not prohibit replication studies, it requires researchers to continue community engagement or develop new research with those who provided consent if they wish to use the data for other purposes. This not only protects descendants and interested and affected persons, groups, and/or parties from the community but furthers opportunities for collaboration.

This approach of obtaining consent for community-engaged scholarship will require restructuring current funding pipelines to make funding available for pre-research consultation and study development, rather than after all permissions are implemented. It also requires compromise and consideration that extend beyond the study, including a willingness to fund products that offer tangible benefits to communities, and acceptance that outcomes may differ from initial predictions. More broadly, funding agencies and their representatives within the scientific community (e.g. reviewers, employers) must recognise that the work of consultation and provision of resources to enable community input is crucial. Particularly in the Global South, descendants, interested and affected persons, groups, and/or parties from local communities, as well as local academic collaborators, often do not have the same resources (e.g. access to libraries, internet, computers, telephones, or even electricity) than their counterparts in the Global North. Lack of capacity produces investment imbalances in the research and limits the roles potential collaborators can occupy before the work even begins^66^.

We argue that consent is both possible and important for aDNA studies for reasons of research integrity and enrichment. We have provided a step-by-step guide for how this process may occur in both the consultation and execution phases of research. We recognise there is a multitude of challenges to obtaining consent that require disciplinary, institutional, regulatory, administrative, and funding modifications for long-term implementation, and see this as an opportunity for researchers, descendants, and other interested parties to co-develop mutually beneficial research. Unfortunately, some researchers may feel scientific robusticity or objectivity will be compromised through some forms of community engagement and inclusion, or with data access models that prioritise community wishes.

We acknowledge that obtaining consent and increasing partnership can slow down research and can be difficult, time-consuming, and emotional work. It is an important way to guard against extractive science and to extend the social benefit of research beyond science. It provides a sense of integrity beyond the study, upholding our commitments to those we study and the people who represent them. The concept of proxy informed consent in aDNA research can be an important element of a discipline-wide set of standards to which we willingly commit and hold one another, even in the absence of legislated mandates. By taking these steps, we have the potential to transform our research approach from studying people to collaborating with and learning from their successors, prioritising research integrity, and enriching aDNA research outcomes.

### Reporting summary

Further information on research design is available in the Nature Portfolio Reporting Summary linked to this article.

### Supplementary information

Supplementary Information

Reporting summary
