# Supplementary material for: Informed proxy consent for ancient DNA research
Source: Commun Biol. 2024 Jul 4;7:815. doi: 10.1038/s42003-024-06413-0 (PMC11224335; doi:10.1038/s42003-024-06413-0)
Supplement: Supplementary file 1 — Supplementary Information [file 42003_2024_6413_MOESM1_ESM.pdf]

Permission to undertake analysis of archaeological remains *Date: _______________*

_________________________ PROJECT POTENTIAL ANALYSES

Initial if Desired	Method	Questions Answerable	Likelihood of Results	Materials Required
☐	DNA Analysis	1. What is the biological sex of the individual?<br>2. What are the population genetics of the individual? Were they a part of the ________ group?<br><br>*Detail other potential questions of interest.*	*Detail which elements were found (e.g., petrous), and if DNA retrieval is therefore likely or unlikely.*<br><br>*Detail reference collections, DNA preservation and likelihood of finding genetic matches at the desired level of interest.*	1. Up to 3 g of bone.<br>2. Sediment sample taken from inside the mortuary feature.<br>DESTRUCTIVE
☐	Protein Analysis	1. What is the biological sex of the individual?	Likely to produce results due to tooth enamel durability	1. Surface etching of tooth enamel (up to 100mg).<br>SEMIDESTRUCTIVE
☐	Bio-geochemical Isotopic Analysis	1. What kind of foods did this person eat (nitrogen and carbon isotopes)? *Also detail region-specific examples of answerable questions.*<br>2. Did this person live in the region their whole life, or did they migrate from a different region (strontium, oxygen, lead isotopes)?	*Detail likely quality of preservation of collagen and enamel.*<br><br>*Detail availability of existing isoscape that will provide reference maps that could yield answers.*	1. Up to 4 g of bone or up to 50mg of tooth enamel (*detail specifics based on preservation*)<br>2. Surface etching of tooth enamel (up to 100mg). 1st and 3rd molars best.<br>DESTRUCTIVE or SEMIDESTRUCTIVE
☐	Tooth Micro-wear Analysis	1. Did this person eat mainly crops or wild foods?<br>2. How did the food they eat affect their teeth?	Likely to produce some results on general dietary patterns	1. Many teeth studied under microscope, with images<br>NONDESTRUCTIVE
☐	Radio-carbon Dating	1. How long ago did this individual die?	Likely to produce results if individual is <50,000 years old. *Detail any specific circumstances that add uncertainty.*	1. Up to 4 g of bone or 500mg of tooth enamel.<br>DESTRUCTIVE
☐	Paleo-pathological Analysis	1. Did this individual suffer from diseases or skeletal stress during their lifetime?	Likely to produce results. *Detail any issues of preservation that may interfere or limit the kinds of pathologies than can be inferred.*	1. Many bones cleaned and studied under microscope<br>NONDESTRUCTIVE
☐	Micro-scopic Bone Surface Analysis	1. What happened to the person after they died?<br>2. Is there evidence of trauma showing manner of death?	Likely to produce some results, but manner of death difficult. *Detail circumstances of preservation and missing elements.*	1. Many bones cleaned and studied under microscope<br>NONDESTRUCTIVE

I, ___________________ (print name), stakeholder and ___________ authority responsible for the human remains found and excavated at __________ in ____ of 20__, give my willing consent for _________________ to undertake **only** the analyses for which my initials appear above. Analyses I do **not** wish considered I have indicated with the word "NO".

Some of these analyses may be done in collaboration with other researchers possessing the necessary equipment and expertise. My signature and that of a witness are below. Desired conditions are printed below and detailed as attached.

STATEMENT OF RISKS AND AGREEMENT

With respect to the analyses described on Page 1 of this document:

I understand that some of the results of the desired analyses may be inconclusive or have unanticipated results.

I understand that ______________ will pay all associated costs of permits, transport, and analysis for the desired analyses.

I understand that some of these analyses will take place in scientific laboratories selected by ______________________.

I understand that some of the selected choices may result in destruction of up to 5g of human bone and/or tooth enamel.

I understand that the __________________ will seek additional permissions if they or their collaborators desire to produce academic publications and/or presentations about specific results.

I understand that ____________ will provide an update on results or pending analyses one year from the date of signature.

I understand that I can revoke this permission in writing at any time.

All results and data from future studies remain the property of ____________________________________.

While results of the analysis are pending, the remains will be stored at the following location:

____

At the conclusion of the study, all samples and data will be stored as detailed below.

Additional conditions agreed at the time of signing (supporting details in attached documents):

____

____

____

____

____

____

Signature:

Title and Printed Name: __________________________

Address: ______________________________________

Phone number: _________________________________

Email address: _________________________________

Date: ___ (day) of _______ (month), 2023

Witness Signature:

Title and Printed Name: __________________________

Address: ______________________________________

Phone number: _________________________________

Email address: _________________________________

Date: ___ (day) of _______ (month), 2023
